# Autonomous Machine Learning Algorithm for Stress Monitoring in Concrete Using Elastoacoustical Effect

**DOI:** 10.3390/ma14154116

**Published:** 2021-07-23

**Authors:** Krzysztof Lalik, Mateusz Kozek, Ireneusz Dominik

**Affiliations:** Faculty of Mechanical Engineering and Robotics, AGH University of Science and Technology, Al. Mickiewicza 30, 30-059 Krakow, Poland; kozek@agh.edu.pl (M.K.); dominik@agh.edu.pl (I.D.)

**Keywords:** non-destructive testing (NDT), machine learning, self-excited systems, acoustoelasticity methods

## Abstract

The measurement of stress in concrete structures is a complex issue. This paper presents a new measurement system called a self-acoustic system (SAS), which uses frequency measurements of acoustic waves to determine the condition of concrete structures. The SAS uses a positive feedback loop between ultrasonic heads, which causes excitation to a stable limit cycle. The frequency of this cycle is related to the propagation time of an acoustic wave, which directly depends on stresses in the test object. The coupling mechanism between acoustic wave propagation speed and stress is the elastoacoustic effect described in this paper. Thus, the proposed system enables the coupling between the limit cycle frequency and the stress degree of the concrete structure. This paper presents a machine learning algorithm to analyse the frequency spectrum of the SAS system. The proposed solution is a real-time classifier that enables online analysis of the frequency spectrum from the SAS system. With this approach, an autonomous system for stress condition identification of concrete structures is built and described.

## 1. Introduction

During the operation of any structure or machinery, it is essential to control the stresses in it for the safety of its users. Over the years, many methods have been developed to monitor changes in concrete materials. When selecting a system to monitor the load on concrete, the speed and simplicity of the measurement, the accuracy of the measurement, and the access to the measuring device should be taken into account.

Generally, in the class of non-destructive testing of stress in concrete, two main measurement methods can be distinguished: quasi-strain gauge methods and ultrasonic methods [1]. Quasi-strain gauge methods consist of attaching a measuring element to the material being tested, which is elongated along with the material being tested [2]. The measuring element uses various physical phenomena to measure its elongation indirectly. In this aspect, a very interesting solution is the use of carbon nanotubes (CNTs) described in [3,4,5,6,7,8]. In [9], cementitious sensors were realised in the form of electrostatically composite carbon nanotube (CNT)/carbon nanotube (NCB) fillers and embedded on concrete columns to develop smart structural stress sensors. In [10], a sensor is described in which a cement composite is filled with multi-layered carbon nanotubes, whose piezoresistive properties enable the detection of mechanical stresses. The same class of solutions also includes those presented in [11,12,13]. These papers indicate that the working principle is to use embedded fibre optic sensors to monitor strain and temperature. In these works, the ability to evaluate the condition of concrete structures with an embedded fibre optic Bragg grating has been experimentally confirmed.

All extensometry techniques are commonly used methods in laboratory conditions to determine the deformation state of a material. In some cases, it is also possible to apply them to in-field techniques. Unfortunately, very often, all the extensometer sensors have to be attached to an unstressed structure. As an incremental technique, they can only indicate the change in stress relative to the sticking condition, not an absolute value. The solution to these problems is the second class of sensors—ultrasonic sensors. Ultrasonic solutions usually utilise piezoelectric ultrasonic heads [14,15,16]. In active ultrasonic systems, monitoring systems use acoustic waves. These have high frequencies from tens of kilohertz to several megahertz. Unfortunately, concrete has a random, multiphase and heterogeneous structure. This causes the received ultrasonic signals to be difficult to interpret and complex in nature. Additionally, in concrete, due to aggregate, acoustic waves in the megahertz range cannot be used because the granular structure of the aggregate results in sound artefacts. In practice, ultrasonic waves of about 250 kHz are typically used. There are also heads using magnetostriction [17,18] or electromagnetic transducers [19,20]. The common disadvantage of these solutions based on acoustic spectrum analysis is a quite serious difficulty in interpretation. Hence, a very interesting solution is the one presented in [21,22,23]. They are based on measuring the change in the acoustic wave propagation velocity associated with the elastoacoustic effect. In these methods, the elastoacoustic coefficient is first determined for a given concrete material, and then the wave propagation velocity between the ultrasonic heads is measured. By knowing the elastoacoustic coefficient and experimentally measuring the wave propagation speed, the stress in the test material can be determined directly. However, this method requires relatively high signal sampling rates and quite high experimental requirements.

The proposed self-excited acoustical system (SAS) also uses the elastoacoustic effect indirectly, but in addition, due to the use of positive feedback, it is more robust to noise [24,25]. The SAS system utilises the self-excitation effect. However, it is rarely desirable due to the possibility of destroying the component being tested, but sometimes used in nanoscale measurement systems [26,27,28]. This system has been successfully applied in civil engineering [29,30], measurement of metallic structures [31,32], mining [33], ceramic, and concrete structures [34,35,36,37].

This paper presents a neural frequency spectrum interpretation system for SAS based on machine learning. The proposed algorithm is designed to identify stresses from the obtained limit cycle spectrum of a self-excited system.

## 2. Methodology

**Figure 1:**
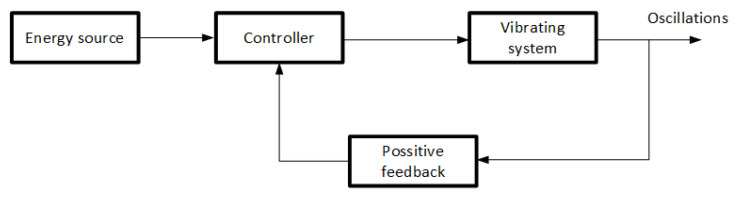
Block diagram of self-excited system.

In the case of self-excited oscillations, the excitation occurs due to the interaction of the system’s internal components. It is a fundamental difference from forced or parametric vibration. Both amplitude and frequency do not depend on the initial state of motion and its intensity in self-excited systems. The SAS is a self-excited system. It is necessary to look at its structure to understand the idea of the operation. The structure of the system, as shown in Figure 1, allows for the occurrence of nondecreasing periodic oscillations despite the energy loss in the system. The energy source supplies the necessary energy to the oscillating element through a regulator whose opening is controlled by feedback from the oscillating element. Assuming that the amplitude of the vibration increases for some reason, then the signal from the controller decreases, causing the amplitude of the vibrating object to change. Conversely, if the control signal increases, the feedback is determined as positive, informing that the energy in the system is too low.

The primary difference between self-excited vibration and forced vibration is that an external energy source is present in both cases, but it is not externally controllable in self-excited vibration. In forced vibration, the actuator can be controlled in the form of external frequency or amplitude regulation, whereas in self-excited vibration, the system itself decides on how much energy it will take into the system, and the external energy source itself may be constant. An example of such an energy extraction mechanism is the Tacoma Narrows Bridge [38]. In this case, the external energy source was the wind blowing at a constant velocity, and the vibration controller was the bridge itself, or more specifically, the stiffness and structure from which its aerodynamics were derived. Although the wind was not harmonic in character, the appropriate energy application resulting from airflow through the bridge structure led to a catastrophic resonance.

Energy losses are present in any mechanical system. In a self-excited system, energy losses are compensated from an external source, which in some cases can lead to nondecreasing periodic oscillations. Once the system is pushed out of its equilibrium position, the system seeks to reach stability again. For an oscillating system in steady-state equilibrium, the amplitude of its oscillations tends to reach a constant value. A stable limit cycle occurs. An oscillating system out of equilibrium with non-cancelling oscillations can increase its amplitude indefinitely.

According to the principle of energy conservation, the change in energy of a mechanical system is balanced by nonconservative forces, which for a self-excited system means that the sum of these forces is zero. For a nonconservative system with one degree of freedom, this can be defined by Equation (1), determined by [39].

(1) $$m\ddot{x}+h\left(x,\dot{x}\right)+f\left(x\right)=0$$

where *m* is the generalized mass, *x* is the generalized displacement, f(x) is the potential force, and $h\left(x,\dot{x}\right)$ is the nonconservative force. The equation for the total mechanical energy $E_{m}$ of the system can be written in the form (4) by substituting to (1) a relation describing the kinetic energy $E_{k}$ of the system (2) and a relation describing potential energy $E_{p}$ of the system (3):

(2) $$E_{k}=\frac{m{\dot{x}}^{2}}{2}$$

(3) $$E_{p}=\int f\left(x\right)\;dx$$

(4) $$dE_{M}=d\left(E_{p}+E_{k}\right)=-h\left(x,\dot{x}\right)dx$$

Equation (4) shows that if the nonconservative forces $h\left(x,\dot{x}\right)$ are positively definite functions, the total change in mechanical energy in the system is monotonically decreasing, leading the system to reach an asymptotically stable equilibrium point. Otherwise, there is an increase in mechanical energy, i.e., the system becomes excited. This leads to a phenomenon in which the system’s motion tends to deviate from an unstable equilibrium. In self-excited systems, the definition of the function $h\left(x,\dot{x}\right)$, which takes both positive and negative values, results in the change of energy being not monotonic. In such oscillations, quasi-harmonic motion is achieved. In such a case, it should be assumed that the physical process is of a kind where the damping conditions can be separated from the excitation conditions. In this case, the energy loss due to damping $\Delta D\left(A\right)$ and the energy provided by the excitation phenomenon $\Delta E_{osc}\left(A\right)$ over the full vibration period *T* can be determined as functions of the vibration amplitude *A*. This relationship is defined by Equation (5).

(5) $$\int_{0}^{T}h\left(x,\dot{x}\right)\dot{x}\;dt=\Delta D\left(A\right)-\Delta E_{osc}\left(A\right)$$

For the periodic solution of Equation (5), condition in Equation (6) must be satisfied from which the amplitude and frequency of the self-excited oscillations can be determined.

(6) $$\Delta D\left(A\right)=\Delta E_{osc}\left(A\right)$$

The frequency of these vibrations will depend on the system parameters, including the velocity of wave propagation in the cement. This, in turn, depends on the elastoacoustic effect. As proven in [40] with stress change in tested material, the propagation speed of acoustic waves also changes. In the range of elastic stresses, in the medium where the directions of textural anisotropy coincide with the directions of principal stresses and for waves propagating and polarised in the directions of acoustic axes determined by texture and stresses, linear changes in the propagation velocity from the stress [41] are observed. The values of elastoacoustic coefficients depend on the type of material and the interrelationship between the wave propagation and polarisation directions and the stress direction. As a rule, the highest values of coefficients are observed for waves in which the directions of particle vibrations are parallel to the direction of stress. According to [42], this relation is defined by Equation (7):

(7) $$\beta_{ijk}=\frac{\left(V-V_{0}\right)}{V_{0}\sigma_{1}}$$

where: $\beta_{ijk}$ is the elastoacoustic coefficient; indexes i,j,k denote the directions of wave propagation, wave polarization, and stress action, respectively; *V* and $V_{0}$ are phase velocities in the stressed and unstressed medium, respectively; and $\sigma_{1}$ is stress.

The frequency and amplitude of the limit cycle of the self-excited system, therefore, depends on the parameter elastoacoustic coefficient and the resulting change in the propagation time of the acoustic wave in cement, which, in turn, depends on the stresses. Therefore, it is possible to determine the stress indirectly on the material being tested by measuring this frequency.

The methodology of the intelligent system is to test a sample made of a known material using an external measurement system and SAS to generate a learning set. The learning set is then used to train an artificial neural network based on a machine learning algorithm. This network should analyse the frequency spectrum and identify the stress in systems that do not have a reference measurement system.

**Figure 2:**
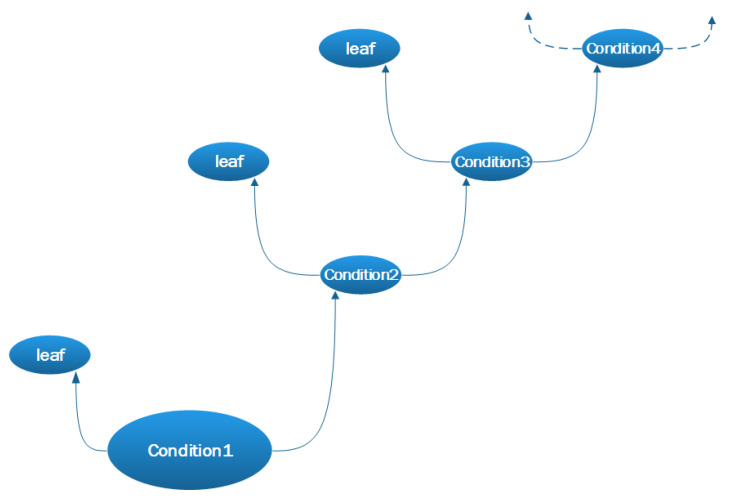
Decision tree for the fine tree algorithm.

The proposed machine learning algorithm is the fine tree, which generates small leaves with a very flexible response function. The decision tree builds regression models in the form of a tree structure (Figure 2). This approach decomposes the data set into smaller and smaller subsets, while at the same time, the associated decision tree is incrementally developed. The result is a tree with decision nodes and leaf nodes. A decision node may have two or more branches. Each branch is responsible for the values for the attribute under study. A leaf represents a decision for a computational objective. The highest decision node in the tree corresponds to the best predictor. The described algorithm thus finds the best solution to the given problem.

A fine tree with many small leaves is usually very accurate on training data. However, in the fine tree algorithm, the results may not show comparable accuracy on an independent test set. The disadvantage may be that a tree with many leaves tends to overfit.

The regression cost function *S* is constructed according to Equation (8). This form causes it to find the most consistent branches or branches with a similar number of responses. It ensures that the batch data follow a specific path.

(8) $$S=\sum_{i=1}^{n}{\left(y_{i}-{\hat{y}}_{i}\right)}^{2}$$

where $y_{i}$ are training data, and ${\hat{y}}_{i}$ is prediction.

Now, the decision tree will begin to split by considering each feature in the training data. The average response of the training data inputs from a given group is taken as the prediction for that group. The above function is applied to all data points, and the cost is calculated for all candidate splits.

(9) $$G=\sum_{i}^{n}\left(p_{i}^{k}\left(1-p_{i}^{k}\right)\right)$$

where $p_{i}^{k}$ is the proportion of inputs of the same class present in a given group.

The concentration measurements of the distribution of the random variable *G*, expressed by Equation (9), allow us to determine how good a split is by how mixed the response classes are in the groups formed by the split. Ideal class purity occurs when a group contains all inputs from the same class: in this case, $p_{i}^{k}=1$. A node having a class split in the 50/50 group has the worst purity. By using the regression cost function, the machine learning algorithm is implemented.

## 3. Measurement Stand

The test object was embedded in a steel frame. The test item was a concrete beam of length L = 1000 mm and cross-section 150 mm × 150 mm with density 600 kg/m${}^{3}$. The beam was made of Portland cement with the parameters shown in Table 1. The concrete beam was not reinforced. The aggregate was about 72% of the total volume of concrete. Fine aggregate—river sand and coarse aggregate—and gravel of 2/16 grain size were used. Sand accounted for more than 30% by weight of the total aggregate.

**Figure 3:**
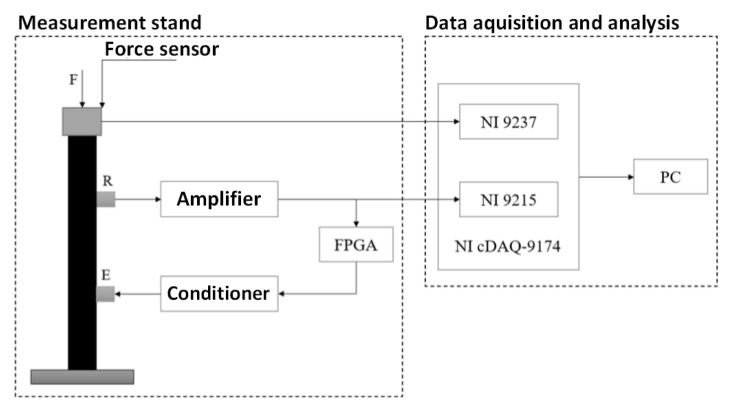
Schematic of laboratory stand with data acquisition module.

A block diagram of the laboratory bench is shown in Figure 3.

A hydraulic cylinder is placed between the beam and the frame. Thus, the beam is put under compression. This action creates stress in the material. The load is measured by a load cell between the top of the specimen and the steel frame. An exciter (E) is attached to the beam’s bottom, and an accelerometer IMI 623C01 from EC Systems (Krakow, Poland, Table 2) and receiver (R) are attached to the beam’s top. The attachment of the elements to the beam is realised through threaded coupling heads, inclined at different angles, glued to the side surface of the concrete. The feedback loop contains a conditioner (VibAMP PA-3000, from EC Systems Krakow, Poland) and a signal amplifier. Behind the conditioner, the FPGA (field-programmable gate array) unit is located. The unit processes and filters the signal received from the conditioner and sends the signal to the amplifier. An operator panel is used to operate the FPGA platform, which runs on a workstation with an operating system. Communication from the application level with the FPGA platform is realised via a USB 2.0.

The last element of the workstation is the data acquisition module, which records the SAS system measurement results on the computer using measurement cards. The cards with a 50 kHz sampling frequency are connected directly to the computer via a USB interface. Card NI9215 is an analogue input module for vibration measurement with a measurement range ± 10 V. Card NI9237 is a strain gauge card for reference load system measurement. It includes the signal conditioning required to power and measure up to four bridge-based sensors simultaneously.

The excitation effect is obtained by coupling the ultrasonic heads: emitting (E) and receiving (R). The emitter introduces an acoustic wave into the tested concrete beam. The wave propagates with a velocity depending on the elastoacoustic coefficient and stress in the test specimen. The accelerometer receives the acoustic wave. After conditioning and amplification, the signal returns to the emitter (E). The resulting positive feedback excites the SAS system to a limit cycle whose frequency depends on the wave propagation time and varies with the stress. The FPGA chip can perform a possible filtering function.

The measurement consists of increasing the beam tension from 5 to 50 kN in 5 kN increments using a hydraulic actuator while introducing vibrations to the beam through an exciter, acting as a transmitter. The response of the system is recorded by an accelerometer, which measures the acceleration of the vibrations. The sampling frequency of the signal was 50 kHz.

**Figure 4:**
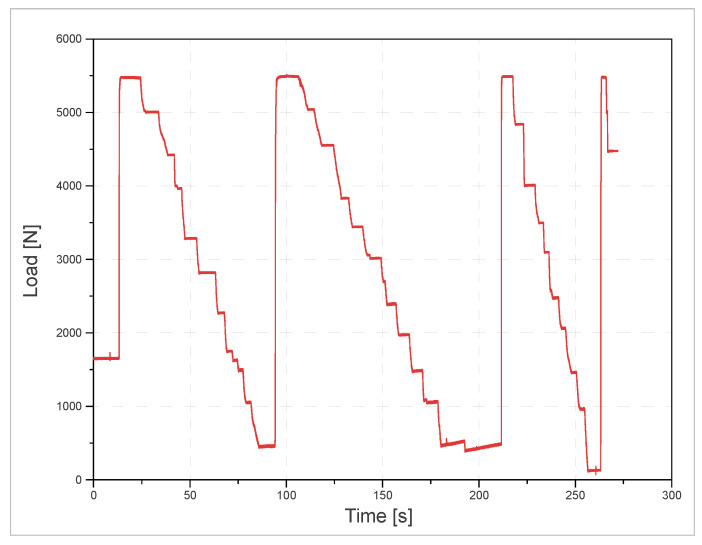
Load curve characteristics in the time domain.

The graph in Figure 4 shows the time domain load characteristics recorded by the force sensor.

**Figure 5:**
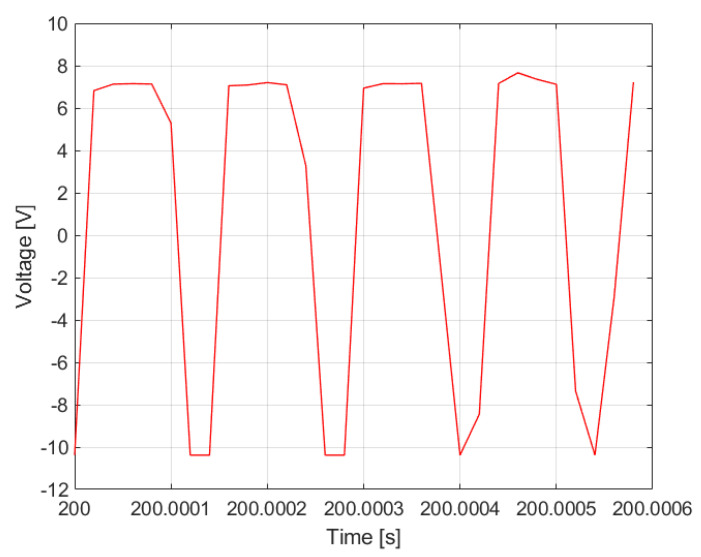
The vibration characteristics of the SAS system.

Figure 5 shows the time domain response of the system recorded during the measurements.

The signal was divided into fragments corresponding to given beam loads, which were then used to determine the frequency spectrum.

**Figure 6:**
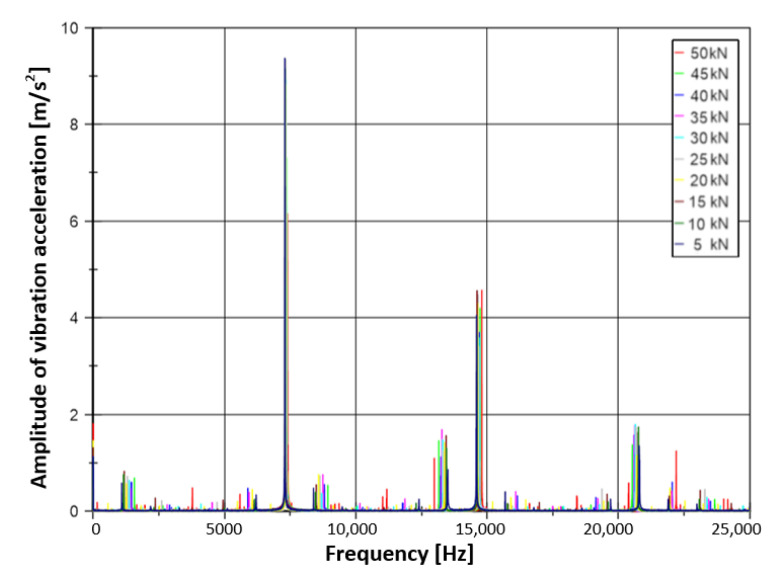
Frequency spectrum diagram of the signal calculated for one load cycle.

Next, to determine the frequency spectrum, a fast Fourier transform was calculated for each signal fragment from all three load cycles. The result of the analysis of one measurement is shown in Figure 6. The signal spectrum is presented in frequency peaks, the height of which corresponds to the amplitude of the individual signal components.

**Figure 7:**
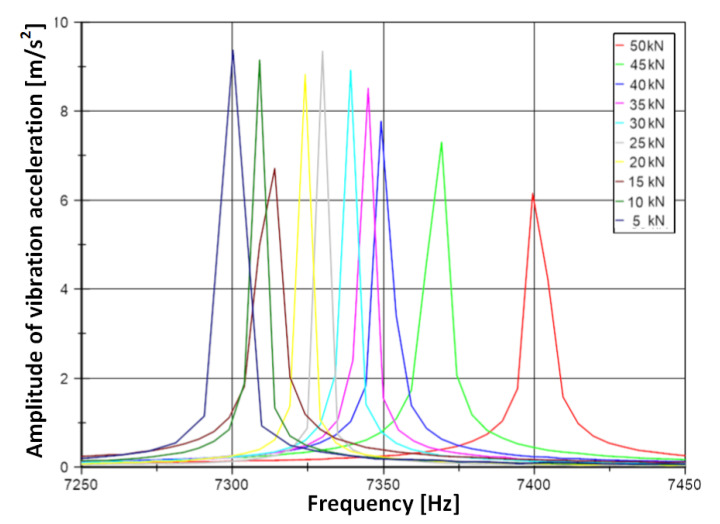
Detailed frequency spectrum of the signal in range 7250–7450 Hz.

The graph in Figure 7 showing the frequency spectrum includes peaks corresponding to frequencies around 7300 Hz. Each of these corresponds to a given load. Figure 7 shows the effect of load on the frequency of vibration. For increasing stress, the frequency has an increasing trend.

The spectrum shown in Figure 6 contains other frequencies in addition to the limit cycle frequency. This is due to the sensor signal saturation and the inhomogeneous concrete structure. Machine learning was used to interpret this spectrum. Its purpose is to analyse the spectrum and answer for what load the spectrum was recorded.

## 4. Results

The learning data were aggregated into two vectors. The load vector came from a reference force sensor. The second vector determined the vibration of the self-excited system. Both vectors were divided into 1 s period intervals, and the average value of each interval was calculated for the load. Thus, a learning set was obtained.

The data were divided into windows of 1 s length and a sample number of 50,000. The number of samples was chosen based on the experience of the research team. A smaller number of samples could have made the spectrum peaks significantly wider, making it difficult for the neural network to interpret the results. The vibration measurement itself was at 50 kHz, while the neural network operated at 1 Hz. It means that the network created a load prediction every 1 s. It is possible to force the response of the neural network at longer time intervals. On the other hand, the time response shortening is restricted by the limitations of the Fourier transform.

**Figure 8:**
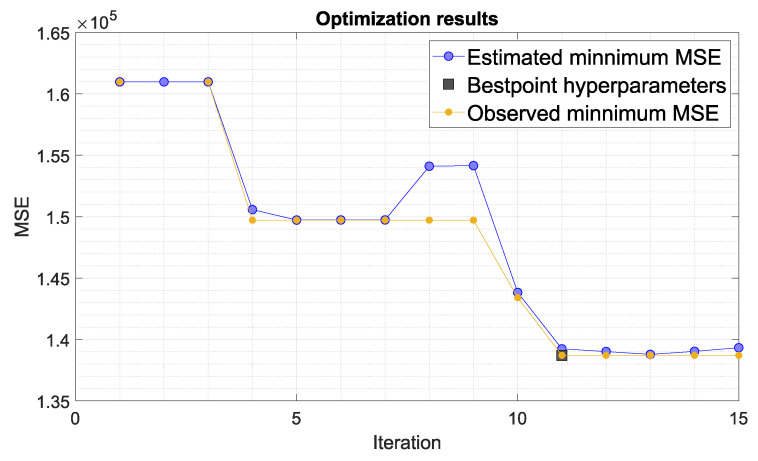
Optimization of the hyperparameters of the neural algorithm.

The optimisation aims to find the point that minimises the objective function for the fine tree algorithm. In the context of hyperparameter selection, the point is the set of hyperparameter values, and the objective function is the loss function, or mean square error. Figure 8 shows the network learning process. Each light blue point corresponds to an estimate of the minimum MSE (mean square error) for all sets of hyperparameter values tried for the current iteration. Each dark blue point corresponds to the observed minimum MSE computed for this optimisation. Noteworthy is the high convergence of the optimisation process, which already satisfies the learning termination condition for 11 iterations.

**Figure 9:**
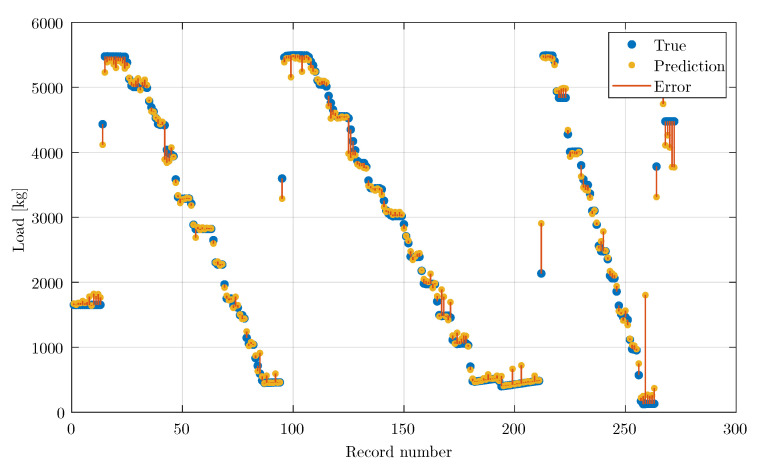
Network learning results.

Figure 9 shows the network learning results. The tested cement beam was unloaded and loaded three times. The yellow colour indicates the load prediction by the neural network, and the blue colour indicates the measurement from the reference system. The highest network error occurred when the load was dynamically changed due to the aggregation methodology of the learning set. The spectrum was created from a waveform with a duration of 1 s. Several frequency changes in the spectrum were evident for dynamic load changes. A solution to this problem may be to decrease the time from which the spectrum is obtained or conduct the tests at a lower load dynamic range.

**Figure 10:**
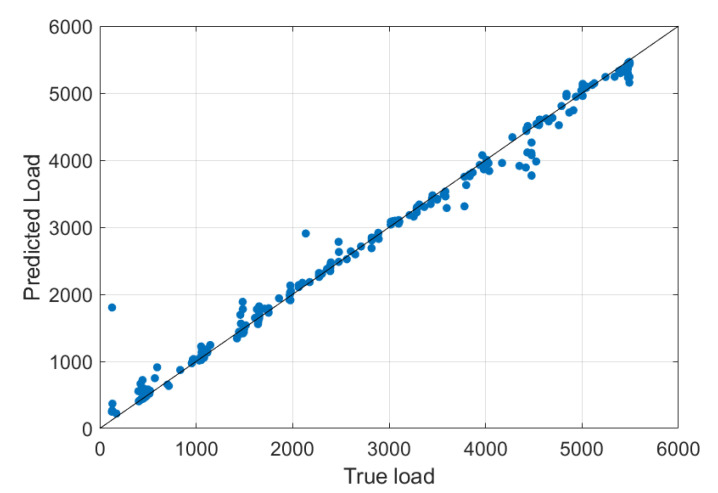
Correlation diagram for machine learning.

Figure 10 shows a correlation diagram indicating the direction and strength of the correlation relationship between the prediction data and the actual observations. The points of the tested correlation cluster along the straight regression line, taking a cigar-like shape, indicating that the relationship’s strength is significant, and thus the excellent quality of the machine learning algorithm. The RMSE (root mean square error) value was 379.18 (N).

**Figure 11:**
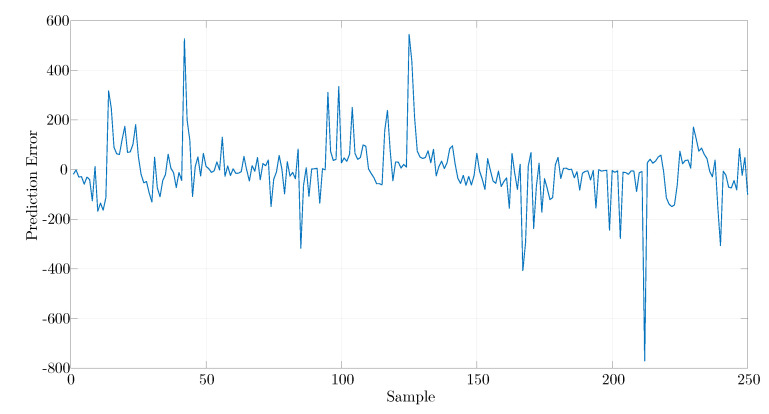
Prediction error for the learning data set.

For the learning set, an error analysis was performed for each element of the learning set shown in Figure 11. The normalised RMSE was 0.01759.

In the next step, the network was trained for three cycles of unloading and reloading in the range of 0–5 kN. Given that the elastoacoustic coefficient is different for different densities of concrete [43], the network had to be learned for each sample separately. The sample dimensions were identical each time as in the baseline experiment. All unloading and reloading cycles above 3 served as the verification set. The learning results are shown in Table 3. The study proved the effectiveness of the learning and the applicability of artificial neural networks in frequency spectrum analysis of the SAS system.

## 5. Conclusions

The elastoacoustic effect is a well-documented way to measure absolute stresses in concrete. The SAS, self-exciting acoustic system, takes advantage of this effect and the self-excitation effect. Unfortunately, a highly detailed identification of the material parameters being tested is required to quantify the absolute value of the stress. In this case, the use of machine learning allows for a quick interpretation of the results. It is necessary to experiment for each concrete density because changing the concrete density causes a change in the elastoacoustic coefficient and consequently a change in the frequency of the self-excited system for the same stresses. Nevertheless, it was shown in the course of the research that the machine learning algorithm:

- Is an effective tool to interpret the frequency spectrum of the SAS system even when there are artefacts on this spectrum related to wave reflections at grain boundaries and the existence of additional noise;
- Can be used for concretes of different densities, and its error determined by normalised RMSE does not exceed 0.0253.

The main feature of an SAS system equipped with a machine learning (ML) algorithm is that it is unnecessary to know the elastoacoustic coefficient. The system equipped with a strain gauge in the lab learns how the frequency spectrum looks for each cement. In the next step in industry conditions, the SAS system equipped with ML predicts the current load of the sample based on the frequency spectrum. Due to the use of ML, it is not necessary to determine the elastoacoustic coefficient every time, which is time-consuming and requires high measurement frequencies. In the course of further work, it will also be possible to reverse train the network. This idea will be based on the fact that the ML network will determine the elastoacoustic coefficient from the spectrum if the load of the tested material is known. It will be the opposite process to the one presented in this paper. Additional training of the network and measurement data from a more comprehensive representation of different concretes will be required.

The study was conducted for an orthogonal head application direction relative to the stress direction. Using machine learning for further investigation will be essential to determine the feasibility of evaluating the stress direction in the concrete and rock materials. Research is being conducted to determine the feasibility of using SAS to analyse stresses by inputting an acoustic wave and receiving it through the ceiling anchoring.

## Figures and Tables

**Table 1:** Chemical specimen composition of the cement sample.

Compound	SiO${}_{2}$	CaO	MgO	Fe${}_{2}$O${}_{3}$	Al${}_{2}$O${}_{3}$	Na${}_{2}$O	K${}_{2}$O	SO${}_{3}$
Content	21.7	65.2	1.3	3.2	5.5	0.34	0.49	2.3

**Table 2:** The Piezo-accelerometer IMI 623C01 specification.

Sensitivity	(±5%)100 mV/g (10.2 mV/(m/s${}^{2}$))
Frequency Range	(±3 dB) 48 to 900,000 cpm (0.8 to 15,000 Hz)
Sensing Element	Ceramic
Measurement Range	±50 g (±490 m/s${}^{2}$)

**Table 3:** Machine learning algorithm results for different concrete densities.

No.	Mean Density $\rho_{0}$ (kg/m${}^{3}$)	Number of Load/Unload Cycle	Normalised RMSE
1	381	9	0.0124
2	457	11	0.0253
3	507	8	0.0149
4	650	5	0.0112
5	704	12	0.0214

## Data Availability

Data sharing is not applicable to this article.

## Abbreviations

The following abbreviations are used in this manuscript:

SAS	Self-excited Acoustical System
NDT	Non-Destructive Testing
CNT	Carbon NanoTubes
FPGA	Field-Programmable Gate Array
MSE	Mean Square Error
RMSE	Root Mean Square Error
ML	Machine Learning
